# Artificial Intelligence-Empowered Doppler Weather Profile for Low-Earth-Orbit Satellites

**DOI:** 10.3390/s24165271

**Published:** 2024-08-14

**Authors:** Ekta Sharma, Ravinesh C. Deo, Christopher P. Davey, Brad D. Carter

**Affiliations:** 1 Artificial Intelligence Applications Laboratory, School of Mathematics, Physics and Computing, University of Southern Queensland, Springfield, QLD 4300, Australia; ekta.sharma@unisq.edu.au (E.S.); christopher.davey@unisq.edu.au (C.P.D.); 2 Centre for Astrophysics, University of Southern Queensland, Springfield, QLD 4300, Australia; brad.carter@unisq.edu.au

**Keywords:** Doppler rate, Doppler dhift, LEO, satellite, artificial intelligence, power efficiency, LoRa

## Abstract

Low-Earth-orbit (LEO) satellites are widely acknowledged as a promising infrastructure solution for global Internet of Things (IoT) services. However, the Doppler effect presents a significant challenge in the context of long-range (LoRa) modulation uplink connectivity. This study comprehensively examines the operational efficiency of LEO satellites concerning the Doppler weather effect, with state-of-the-art artificial intelligence techniques. Two LEO satellite constellations—Globalstar and the International Space Station (ISS)—were detected and tracked using ground radars in Perth and Brisbane, Australia, for 24 h starting 1 January 2024. The study involves modelling the constellation, calculating latency, and frequency offset and designing a hybrid Iterative Input Selection–Long Short-Term Memory Network (IIS-LSTM) integrated model to predict the Doppler weather profile for LEO satellites. The IIS algorithm selects relevant input variables for the model, while the LSTM algorithm learns and predicts patterns. This model is compared with Convolutional Neural Network and Extreme Gradient Boosting (XGBoost) models. The results show that the packet delivery rate is above 91% for the sensitive spread factor 12 with a bandwidth of 11.5 MHz for Globalstar and 145.8 MHz for ISS NAUKA. The carrier frequency for ISS orbiting at 402.3 km is 631 MHz and 500 MHz for Globalstar at 1414 km altitude, aiding in combating packet losses. The ISS-LSTM model achieved an accuracy of 97.51% and a loss of 1.17% with signal-to-noise ratios (SNRs) ranging from 0–30 dB. The XGB model has the fastest testing time, attaining ≈0.0997 s for higher SNRs and an accuracy of 87%. However, in lower SNR, it proves to be computationally expensive. IIS-LSTM attains a better computation time for lower SNRs at ≈0.4651 s, followed by XGB at ≈0.5990 and CNN at ≈0.6120 s. The study calls for further research on LoRa Doppler analysis, considering atmospheric attenuation, and relevant space parameters for future work.

## 1. Introduction

It is estimated that 24% of catalogued items in space are satellites, with less than one-third being operational [1]. Furthermore, roughly 18% of items are expended in upper stages and components associated with missions. The increasing proliferation of objects in Earth’s orbit poses a considerable obstacle to space missions and infrastructure. There is a growing interest in existing radio signals for bi-static or multi-static radar applications to improve monitoring capabilities. Radiofrequency systems, specifically radar technology, are effective solutions for monitoring space objects, particularly those in LEO. RF sensors can function regardless of lighting or weather conditions, making them reliable tools for observing and tracking objects in space. Researchers in [2] have designed an in-depth analysis of the Multibistatic Radar architecture. They focused on the system design, and signal processing considerations associated with a radar used in tracking and space monitoring applications. The compact tracking RADAR system used to monitor celestial bodies in Low Earth Orbit was developed individually [3].

LEO satellites have shown great promise as a foundational infrastructure for advanced global wireless networks, offering increased data rates and improved connectivity. Given their reduced transmission delay and adaptable service options, LEO satellites are typically favoured for enhancing and expanding the capabilities of future terrestrial communication networks [4]. Nevertheless, the obstacles presented by prompt transmission rates, negligible latency, and widespread connectivity persist [5]. Furthermore, as a result of advancements in diverse applications, these criteria must be upheld even in challenging conditions, such as environments with high levels of mobility. There are often substantial channel fluctuations in these settings, which result in decreased communication efficiency [6]. LEO satellites maintain a velocity significantly higher than the rotation speed of the Earth to prevent reentry into its atmosphere due to gravitational forces. The observed phenomenon leads to a significant change in the frequency offset of the signal being transmitted, commonly referred to as the Doppler shift, which varies periodically within the satellite’s timeframe. The Doppler rate symbolises the velocity at which these fluctuations take place, determined by the rate of Doppler shift changes over a specified period of time. Regrettably, this presents significant obstacles to the functionality of LoRa receivers at the physical layer [7]. LoRa modulation is a modulation scheme derived from the chirp-spread-spectrum (CSS) modulation technique [8].

Traditional methods for Doppler shift estimation have been proposed in previous literature, such as the channel autocorrelation coefficient function (ACF) [9], phase difference tracking [10], cyclic prefix [11], and maximum-likelihood estimation [12]. In [13], a linear frequency modulation (LFM) preamble-based Doppler estimation method was introduced. While these methods are effective, they require extensive sampling and can be challenging to implement packet-by-packet. Additionally, they are sensitive to additive white Gaussian noise (AWGN), reducing accuracy in low SNR environments. Authors in [14] have presented a novel Doppler detection framework designed for blind opportunistic navigation with the utilisation of signals from LEO satellites.

Artificial intelligence (AI) offers us strategies to address these challenges, thanks to its advancements and use in communications. There has been a growing interest in deep learning (DL) algorithms for addressing intricate challenges across various communication sectors. However, limited research has been conducted on the estimation of Doppler effects for LEO satellites using deep learning techniques. The use of deep learning algorithms for predictive channel modelling and advanced multibeam precoding techniques in complex satellite communication (SatCom) environments was performed by [15]. The analysis of channel forecasting and the hybrid beamforming in an LEO satellite’s massive Multiple-Input Multiple-Output (MIMO) system was researched by [16]. Authors in [17] used advanced deep learning techniques to detect overlaid control signals in LEO Multiple-Input Multiple-Output (LEO-MIMO) systems.

In artificial intelligence technology, the deep neural network (DNN) stands out for processing vast amounts of data and tackling intricate nonlinear challenges, thanks to its data-driven nature. Specifically in the time-series issues, the gated recurrent unit (GRU), or recurrent neural network (RNN), demonstrates impressive predictive abilities. The study [18] focuses on using CNN-assisted techniques for enhancing channel and carrier frequency offset estimation in high-altitude platform station (HAPS)–LEO communication links. Initially, efforts are directed towards alleviating channel distortion and carrier frequency offset, including residual Doppler effects, through a novel estimator based on Convolutional Neural Networks. Subsequently, the data transmission rate is improved by enhancing the spectral efficiency with multiple access methods. The heightened focus on the DL benefit is attributed to their superior capability to extract several features that conventional methods struggle to achieve. This paper [19] introduces a CNN-based model for estimating Doppler shifts in LEO SatCom systems. The model uses deep learning to extract channel features and reduce errors in predicting Doppler shifts. Numerical results demonstrate the model’s accurate estimation capabilities. The authors’ [20] architecture proposed a deep neural network to estimate timing deviation and frequency offset in high-mobility multicarrier transmission using CNN for extracting hidden signal features.

To leverage the benefits of deep learning technology, we have developed a sophisticated model for low-Earth-orbit (LEO) constellation satellites. Our model evaluates the connectivity between satellites and ground stations, calculates latency, and incorporates a hybrid and computationally efficient model integrating Iterative Input Selection and Long Short-Term Memory Network (IIS-LSTM). This innovative model aims to forecast the Doppler profile for LEO satellites. Our comprehensive examination takes into account the Doppler shift and Doppler rate to evaluate their effect on packet loss. Doppler shift, otherwise referred to as static Doppler, adversely impacts communication effectiveness when at shallow elevation angles and the furthest reach of the link.

The Doppler rate, or dynamic Doppler, can cause loss of packets at high angles of elevation, especially in the close proximity of the satellite to ground devices, which ultimately leads to a reduction in the link distance. Additionally, the involvement of deep learning (DL) is a rapidly evolving area of study. There has been a noticeable increase in interest and dedication toward exploring low-power and long-range communication networks, such as LoRa, for sensor networks and the Internet of Things. LoRa provides an economical and energy-conserving solution for communication. It forms the basis for terrestrial Low-Power Wide-Area Network (LPWAN) technology, LoRaWAN.

The study conducted by authors [21] explores detecting LoRa signals from a LEO satellite, posing challenges due to Doppler effects and interference. Research by [22] showed the promising results of LoRa technology for satellite IoT, particularly in LEO. Testing demonstrated significant resistance to the Doppler effect, allowing for the utilisation of LoRa technology in orbits exceeding 550 km without any limitations. Below 550 km, the Doppler rate may disrupt communication sessions, with a minute reduction in LEO at 200 km. Therefore, we believe it is imperative to consider the impact of performance communication constraints. These can be the signal-to-noise ratio, bandwidth allocation, and the carrier frequency of utilising LoRa modulation within the framework of the Doppler effect. This can lead to potential sway over the frequency variations, packet loss, and the subsequent disruption of communication pathways. This will play a role in determining the receiver’s susceptibility to this phenomenon. LoRa technology and LEO satellites have also been examined in previous studies, particularly in terms of link budget examination and scalability within networks for enabling uplink connectivity. Studies such as [23] find that link budget calculations suggest that communication up to 2302 km is possible. To ensure a reliable satellite-to-ground 471 MHz link for LEO small satellites, the authors in [24] calculated a link budget for the LoRa satellite with a transmission power of 17 dBm and reception sensitivity of −123 dBm. It is important to address the performance due to Doppler, which presents a significant challenge for LoRa uplink connectivity. Our approach aims to address gaps in the literature, aiming for novelty by accomplishing the following tasks:

- The study examines a space surveillance radar system for tracking and monitoring the trajectories of low-Earth-orbit (LEO) satellites, specifically targeting the Globalstar and International Space Station (ISS) satellites. The analysis utilises two strategically chosen locations, Perth and Brisbane, to ensure comprehensive East–West coverage.
- The study creates models for LEO constellation satellites, assessing satellite–ground-station connectivity, determining latency, and developing a hybrid and computationally efficient model integrating Iterative Input Selection and Long Short-Term Memory Network (IIS-LSTM). This model seeks to accurately predict the Doppler profile for the weather data of LEO satellites with the highest level of precision. We consider both the Doppler rate and Doppler shift to gauge the influence of each on packet loss.
- The IIS algorithm acts as a feature selector to minimise the dimensionality of the input space by selecting the relevant input variables or features that contribute to the model’s performance. The LSTM algorithm acts as the learning model for understanding and predicting patterns.
- The research aims to examine the impact of the capacity constraints of LoRa modulation in response to a significant effect of the Doppler effect caused by the movement of LEO satellites. The investigation involves an evaluation of various communication parameters, such as the spreading factor, altitude of the satellite orbit, frequency offset, and bandwidth of the signal, which affect the receiver’s susceptibility to this occurrence.
- Benchmarking is conducted using the machine and deep learning models—Convolutional Neural Network (CNN) and Extreme Gradient Boosting (XGBoost)—to enhance efficiency and performance.

Based on our current understanding, this study represents a pioneering analysis of the effects of deep learning involving IIS-LSTM on a comprehensive set of parameters about the resilience of LoRa technology to the Doppler effect within a low-Earth-orbit (LEO) environment.

The structure of this paper is as follows: Section 2 provides a vast overview of the Methodology, including the study area and LEO satellite tracking radar system, carrier frequency offset estimation, AI model framework for doppler weather profile analysis, impact of doppler on LoRa satellite and satellite link budget perspective. Following that, the results and discussion are examined in Section 3. Finally, Section 4 and Section 5 present the concluding remarks and suggestions for future research.

## 2. Methodology

### 2.1. Study Area

**Figure 1:**
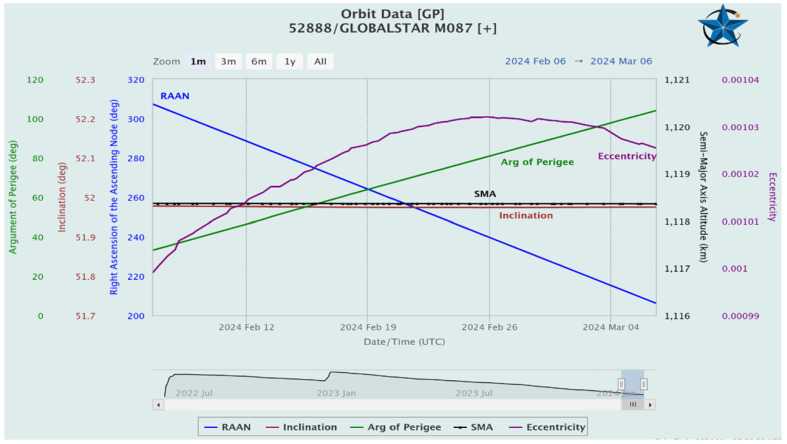
One-month projection for Globalstar satellite with orbit data including altitude (km), eccentricity, right ascension (deg), inclination (deg), argument of perigee (deg), and Date/Time on *X*-axis. Source: CelesTrak.

Two carefully selected Australian locations, Perth and Brisbane, as described in Table 1, were identified for this research study. These stations were chosen due to their advantageous position along an East–West axis. A timeframe of 24 h, starting on 1 January 2024, as illustrated in Figure 1, was considered. This timeframe proved to be instrumental in conducting radar detection simulations and monitoring trajectories, which was an initial essential component of the study. A thorough assessment was carried out to evaluate the connectivity between LEO satellites and the Australian ground stations. This evaluation aimed to determine the visibility of the ground stations from the satellites’ perspective. This analysis aids in pinpointing the satellites that are within the line of sight of the ground stations at any given moment.

### 2.2. LEO Satellite Tracking Radar System

In the initial task, the study examines a space surveillance radar tracking system that employs a low-fidelity model to forecast the projected orbit of each satellite. The satellite scenario object imports a satellite orbit constellation defined within a Two Line Element (TLE) file, a widely used format for storing satellite orbital information [25]. The TLE file containing orbital data for two satellite constellations, Globalstar and International Space Station NAUKA (ISS NAUKA), has been chosen to examine their detection and tracking capabilities. The imported satellite orbits undergo propagation using the Simplified General Perturbations-4 (SGP4) orbit propagation algorithm [26], which is renowned for its accuracy with objects in low Earth orbit. These orbits are used as a reference point for evaluating the radar’s capability of tracking to find the latest satellites. Subsequently, synthetic detection and tracking scenarios are generated through a space surveillance radar simulation. This simulation involves configuring two radar stations with strategically placed fan-shaped radar beams to enhance detection rates by overlapping satellite orbits. Following this, radar detection simulations and trajectory monitoring were carried out for two designated Australian stations, specifically Perth and Brisbane, positioned strategically in an East–West orientation, spanning 24 h commencing on 1 January 2024, as shown in Figure 1. An evaluation of the accessibility between LEO satellites and the Australian ground station was conducted to determine the visibility of the ground station to the satellites. This analysis helps to identify satellites in the line of sight of the ground station at any given moment. Each station is equipped with a radar system tailored to find the LEO satellites, with the following specifications:

- Is capable of detecting a 10 dBsm object at a range of 2000 km.
- Effectively and accurately aligns objects in directions within a 100 m margin of error at a distance of 2000 km.
- Provides 30 degrees in elevation and a range of 120 degrees in the azimuth.
- Is positioned to observe the sky and monitor space activities.

The radar models yielded detections which were used to estimate satellite orbits. A Joint Probabilistic Data Association (JPDA) tracker [27] was chosen for its efficient tracking capabilities and optimal use of computational resources. A tracking filter was established for the tracker, employing a simplified model, like Keplerian integration, rather than SGP4, to track satellite movements. Any inaccuracies in the target’s motion model were addressed through measurement updates and the integration of process noise within the filter.

**Figure 2:**
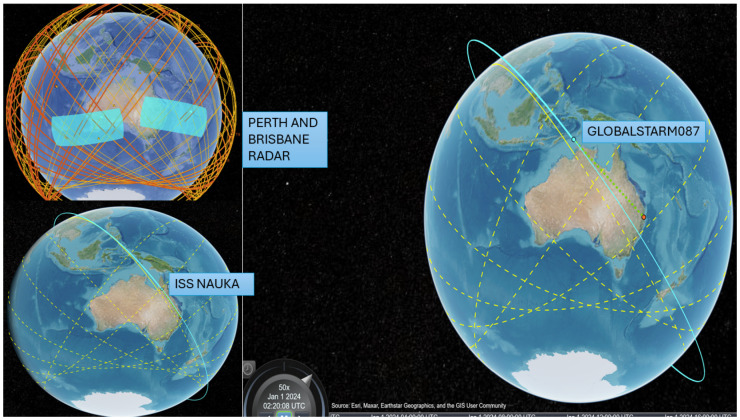
Radar detection of Australian study sites and the anticipated paths of the GlobalstarM087 and ISS NAUKA satellites’ movements on the ground within 12 h. The previous trajectories are shown through continuous yellow lines, and dotted yellow lines distinguish upcoming trajectories.

Subsequently, radar detections were simulated to track satellites, ensuring accurate tracking of each satellite’s position. We generated a comprehensive record of the constellation’s states over 12 h, followed by a simulation of radar detections and track generation. After five hours of diligent tracking efforts, approximately half of the constellation was successfully tracked. It presents a significant challenge to uphold pathways with incomplete orbit coverage, as satellites can remain unnoticed for an extended duration in this arrangement. It is anticipated that if we add further stations for tracking, it will enhance the overall detection ability. The assignment metrics, which evaluate tracking performance by comparing objects, are outlined in Figure 2. We used orbit data from Two Line Element (TLE) files to calculate and visually represent satellite trajectories within our simulation software tool. The study uses the unscented Kalman filter [28] with a Keplerian motion model, setting Alpha to 1, Beta to 0, and Kappa to 0 to ensure robustness for longer forecasting periods. Latency has been attributed to the considerable distance between satellites and the Earth. Furthermore, LEO satellites have been observed to reduce latency as opposed to geostationary (GEO) and medium-Earth-orbit (MEO) constellations, owing to the shorter proximity between the terminal and the satellite.

**Figure 3:**
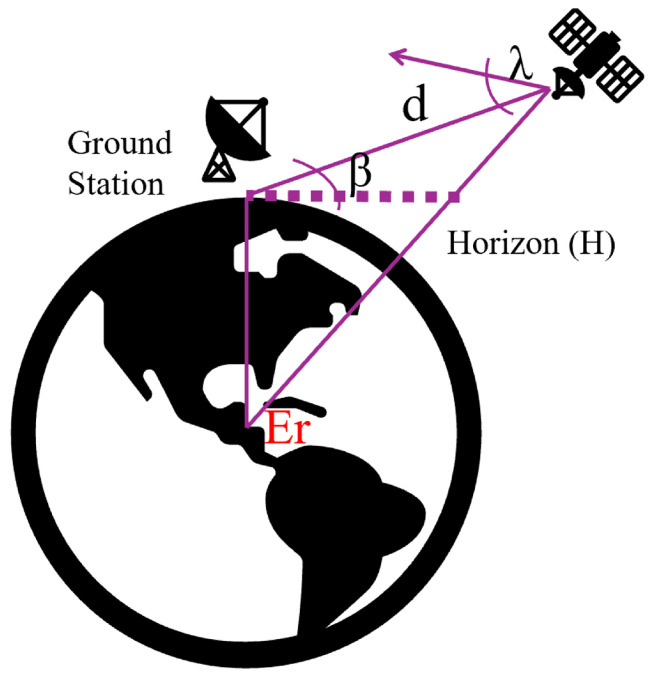
Satellite-to-end-device positioning. E symbolises the elevation angle caused by the sudden oscillations of the LEO satellite, S denotes the slant distance impacting the total distance travelled, and β stands for the elevation angle of satellites.

To effectively analyze the performance of the LoRa Uplink, it is essential to possess a deep understanding of the satellite-to-end-device positioning, as illustrated in Figure 3. Let E symbolise the elevation angle caused by the sudden oscillations of the LEO satellite, let S denote the slant distance impacting the total distance travelled, and let β stands for the elevation angle of satellites. Then, by applying the geometric principles, individuals can ascertain the slant distance (d) through a designated elevation angle (E), and vice versa (as shown in Equation (1)).

(1) $$S=\sqrt{Er\sin^{2}\beta +2H*Er*\sin\beta +H^{2}}-Er\sin\beta$$

The key factors considered in this study for the satellites include bandwidth, application payload, low-data-rate optimisation, spreading factor, elevation angle, average inter-arrival time, loss due to static Doppler shift, loss due to dynamic Doppler shift, and LEO pass duration. Additionally, Figure 1 showcases the names and orbital paths of the satellites. Figure 2 illustrates the anticipated path of the satellite’s movement on the ground within 12 h. It distinguishes between the previous and upcoming trajectories by utilising dotted yellow lines for the latter and continuous yellow lines for the former.

### 2.3. Carrier Frequency Offset Estimation (CFO)

**Figure 4:**
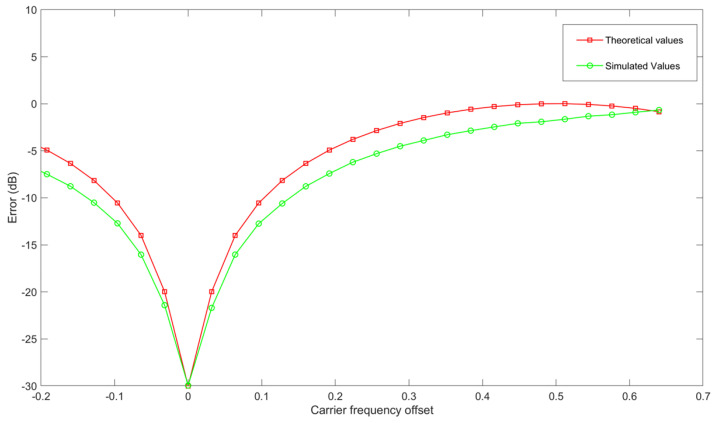
Carrier frequency offset on the *X*-axis with the error in decibels on the *Y*-axis. The red trajectory shows theoretical values, and simulated values are represented in green.

We estimated the CFO and calculated the degradation of signal-to-noise ratio (SNR) resulting from frequency offset in Orthogonal Frequency Division Multiplexing (OFDM) modulation. The data rate per OFDM symbol corresponds to the number of subcarriers for Binary Phase Shift Keying (BPSK). The signal-to-noise ratio (SNR) spans from 0 to 30 dB, with frequency increments occurring at intervals of 10 MHz within the range of −200 to 200 MHz. Figure 4 illustrates the relation between CFO on the *X*-axis and the error magnitude in decibels on the *Y*-axis. The red trajectory shows theoretical values, and simulated values are represented in green.

### 2.4. AI Model Framework for Doppler Weather Profile Analysis

Selecting the radar mode (which can be selected as either “uniform” or “staggered”) and radar wavelength for both satellites, as discussed earlier, synthetic weather data were generated to evaluate the impact of Doppler as outlined by the authors [29]. The inputs required for the data include power levels, spectrum widths, mean Doppler velocity, noise power, number of samples within a coherent processing interval (CPI), sequence length, pulse repetition frequency, list for the staggered sequence (default setting is (2 or 3), samples factor required for window effects (default setting is 10), and the number of realisations.

Without specific guidelines, the data were initially split into a training set comprising 75%, a validation set comprising 15%, and a testing set containing 10%. The models were programmed using Python version 3.7 and MATLAB version R2023a for Windows. We have used MATLAB’s SatCom Toolbox programming languages and utilised freely available open-source libraries such as numpy, pandas, matplotlib, sci-kit-learn, TensorFlow, and optuna. We conducted model training across various architectures, utilising both the Graphics Processing Unit (GPU) and Central Processing Unit (CPU) to assess and compare the efficiency of training and prediction times. The models were constructed using the Windows 10 operating system on an Intel i7 Generation 9 processing unit clocked at 3.7 gigahertz, with 16 gigabytes of memory and an Nvidia 1050 Ti GPU, 4.

Our research involves an in-depth analysis of three unique machine and deep learning architectures through training and assessment using a simulated dataset. We are specifically investigating the effects of different training factors, such as signal-to-noise ratios, quantisation levels, and sequence lengths, on the precision of estimation. Furthermore, we are assessing the ability of each architecture to handle potential receiver scenarios in terms of scalability.

The research entails the creation of intricate models for LEO constellation satellites, analyzing the connectivity between satellites and ground stations, assessing latency, and developing a hybrid and computationally efficient model integrating Iterative Input Selection–Long Short-Term Memory Network (IIS-LSTM). The primary goal of this model is to accurately forecast the Doppler profile for the weather data of LEO satellites with a high degree of accuracy and calculate the time delay, Doppler and frequency shift between the satellites and the ground stations. Our thorough evaluation takes into account both the Doppler rate and the Doppler shift to assess the influence of each on packet loss.

The satellite timeframe for this analysis spans from 1 January 2024, starting precisely at 12:00:00 AM Australian Eastern Standard Time (AEST), for 24 h. The simulation operates at a real-time sampling frequency of 1 min. In this analysis, a C band frequency of 5 GHz has been utilised for measurement purposes. The Globalstar terrestrial band offers a bandwidth of 11.5 MHz, while the ISS NAUKA satellite operates at a frequency of 145.8 MHz.

#### 2.4.1. Globalstar and ISS Satcom Arrangement

Globalstar and ISS have standardised arrays comprising uniform elements such as A antennas, with $A_{y}$ antennas on the *Y*-axis and $A_{x}$ antennas on the *X*-axis.

Each antenna is strategically placed equidistantly with half a wavelength for optimal signal reception and transmission. With one antenna, both satellites provide communication services to a user. Further to the models outlined [30]. If t is the time from the satellite, O is the altitude of the orbit, and O(t) ϵ $w^{A}$ is the channel with obstructions or no obstructions between two transceivers.

Mathematically,

(2) $$O\left(t\right)=\sqrt{\frac{RF}{RF+1}}\bar{O}\left(t\right)+\sqrt{\frac{RF}{RF+1}}\tilde{O}\left(t\right)$$

(3) $$\bar{O}\left(t\right)=e^{k2\pi \left(\bar{f_{ds}}-CF\bar{\mu }\right)}\nu \left(\bar{e},\bar{\varphi }\right)$$

(4) $$\tilde{O}\left(t\right)=\sqrt{\frac{1}{L}}\sum_{l=1}^{L}g_{l\;\cdot \;}e^{k2\pi \left(\tilde{f_{ds}}-CF\tilde{\mu }\right)}\nu \left(\tilde{e},\tilde{\varphi }\right)$$

Below, we mathematically express the equations in the context of our current discussion. Within this framework, we define the following variables for the line-of-sight path:

$\bar{\varphi }$ = azimuth angle,
$\bar{\mu }$ = propagation delay,
$\bar{e}$ = elevation, and
$\bar{f_{ds}}$ = Doppler shift.

For the non-line-of-sight path:

$\tilde{\varphi }l$ = azimuth angle,
$\tilde{\mu }l$ = propagation delay,
$\tilde{e}l$ = elevation, and
$\tilde{f_{ds}l}$ = Doppler shift.

Moreover, we introduce the symbols CF, L, and RF to signify the frequency offset, NLOS paths, and Rician factor. Lastly, the array response vector ν(θ,φ) is characterised as such for our analysis.

(5) $$\nu \left(\theta ,\varphi \right)=\nu_{x}\left(\theta ,\varphi \right)⊗\nu_{y}\left(\theta ,\varphi \right)$$

(6) $$\nu_{x}\left(\theta ,\varphi \right)={\left[1,e^{-\frac{k2\pi asf}{c}}\cos\theta \sin\varphi ,\ldots ,e^{-\frac{k2\pi asf}{c}}\left(A_{x}-1\right)\cos\theta \sin\varphi \right]}^{T}$$

(7) $$\nu_{y}\left(\theta ,\varphi \right)={\left[1,e^{-\frac{k2\pi asf}{c}}\cos\theta \sin\varphi ,\ldots ,e^{-\frac{k2\pi asf}{c}}\left(A_{y}-1\right)\cos\theta \sin\varphi \right]}^{T}$$

(8) $$\nu \in \Delta^{AxSx2}$$

where ⊗ is the Kronecker product, antenna spacing is ‘as’, and c is the speed of light.

#### 2.4.2. The Proposed IIS-LSTM Modelling Approach

The model consists of a hybrid involving an IIS algorithm that functions as a feature selector and LSTM as a deep learning algorithm. IIS streamlines the input space dimensions by identifying the most important input variables (features) that enhance the model’s performance. It identifies the most advantageous attributes within the innovative hybrid integrated model. The channel matrix is transformed into a three-dimensional tensor β with dimensions of

(9) $$F_{n}=\beta \left(W_{m\;\cdot \;}\left(LH_{n-1},NI_{n}\right)+V_{m}\right)$$

β is the input tensor that takes on the appearance of a dual channel. After feature extraction, a component of Recurrent Neural Networks (RNNs), LSTM, was utilised, which possesses the capability to capture long-term dependencies. These models have recently attracted increasing interest. The chief advantage of LSTM networks lies in their ability to consider the sequential progression of events in a forecasting period, rendering them well suited for sequential AI methodologies. In Long Short-Term Memory networks, there are input, output, and forget gates within memory blocks that effectively update and regulate the flow of information in distinct blocks. This unique feature of LSTM is beneficial as it facilitates the continual updating of future predictions.

If $LH_{n-1}$ = last hidden state, $NI_{n}$ = new input, $FG_{n}$ = forget gate, $W_{m}$ = weight matrices, $V_{m}$= bias vector, $ig_{n}$ = input gate, θ(..) = logistic sigmoid activation functions, tanh(..) = hyperbolic tangent. Further to $S_{n-1}$ and $NI_{n}$, LSTM can determine the information that is to be excluded or thrown away from the cell state represented by

(10) $$CS_{n}=\tanh\left(W_{c\;\cdot \;}\left(LH_{n-1},NI_{n}\right)\right)$$

Deciding what information will be stored in the cell state, we need another candidate cell state $CS_{n}$ that is generated and scaled by

(11) $$ig_{n}=\left(W_{i\;\cdot \;}\left(LH_{n-1},NI_{n}\right)+V_{i}\right)$$

(12) $$CS_{n}=\left(F_{n}*C_{n-1}+NI_{n}*C_{n}\right)$$

$CS_{n}$, which is the latest state of a cell, is updated by merging the earlier state of the cell $CS_{n-1}$ and the latest state of the cell $CS_{n}$. The earlier cell is affected by the ‘forget gate’ $FG_{n}$ and the latter by ‘input gate’ $ig_{n}$:

(13) $$\partial_{n}=\sigma \left(W_{\partial \;\cdot \;}\left(LH_{n-1},NI_{n}\right)+b_{\partial }\right)$$

The output process is conducted sequentially, comprising two distinct steps. A newly introduced gate, referred to as the ‘output gate’ $\partial_{n}$, is employed to determine the specific elements of the cell state (the $C_{n}$ that is to be outputted). The activation of the cell state through the tanh function is refined by the application of the ‘output gate’ $\partial_{n}$. The resultant product of this multiplication operation yields the anticipated output, denoted as Sn.

(14) $$LH_{n}=\partial_{n}\tanh\left(C_{n}\right)$$

Benchmarking is performed with two other machine and deep learning models (Table 2), Extreme Gradient Boosting (XGBoost), and Convolutional Neural Network (CNN) which have been covered in our earlier studies [31,32]. With precise calculations for Doppler shift and additional data extracted related to Doppler, we also took care of the trajectory of its variation. The data output for Doppler includes relative velocity and Doppler rate.

**Figure 5:**
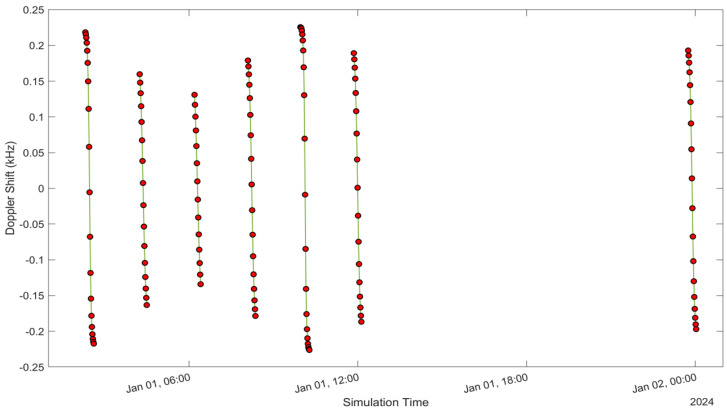
Doppler shift of the Globalstar satellite with initial satellite data.

**Figure 6:**
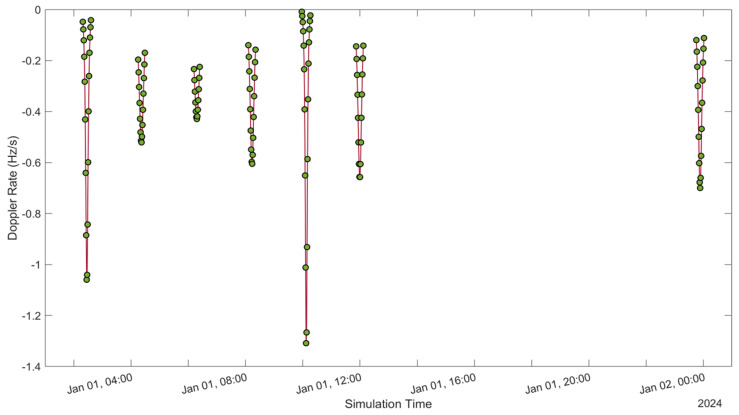
Doppler rate of the Globalstar satellite with initial satellite data.

**Figure 7:**
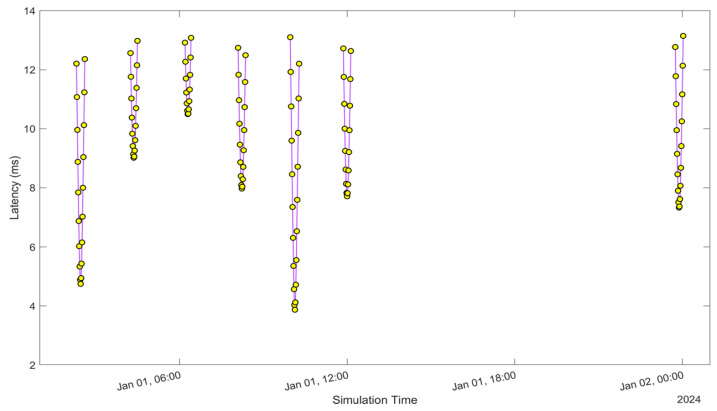
Latency of the Globalstar satellite with initial satellite data.

**Figure 8:**
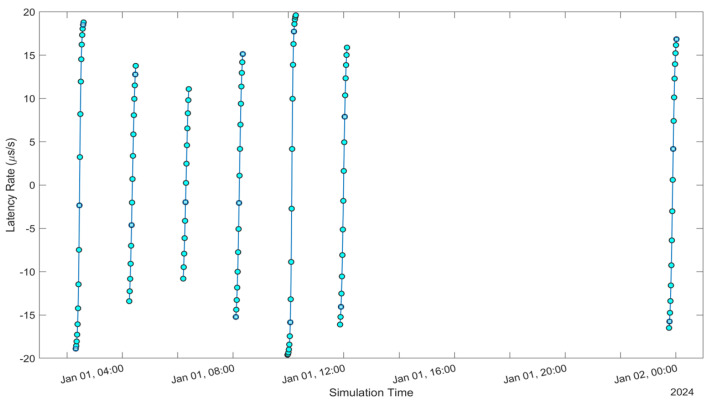
Latency Rate of Globalstar satellite with initial satellite data.

**Figure 9:**
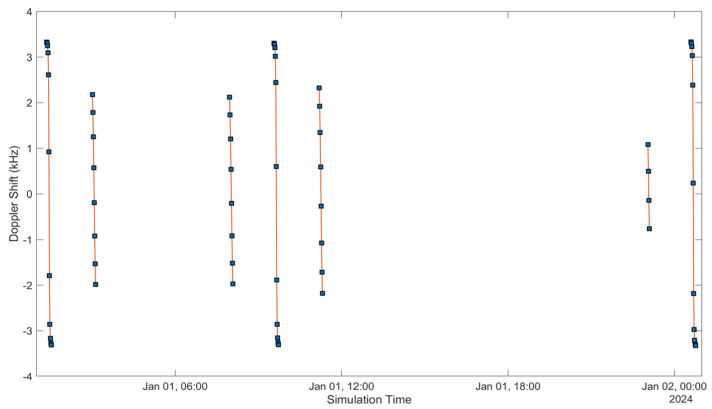
Doppler shift of the ISS NAUKA satellite with initial satellite data.

**Figure 10:**
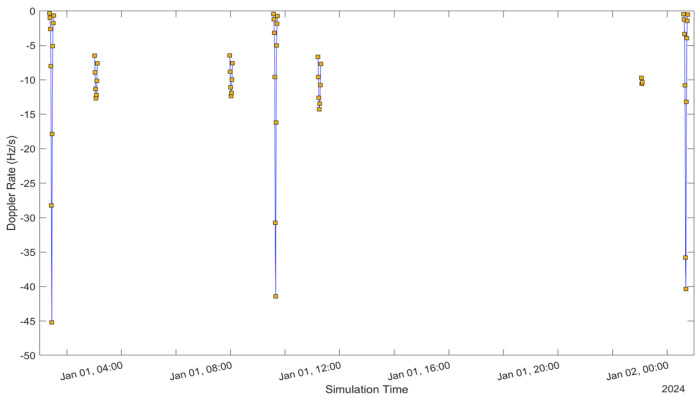
Doppler rate of the ISS NAUKA satellite with initial satellite data.

**Figure 11:**
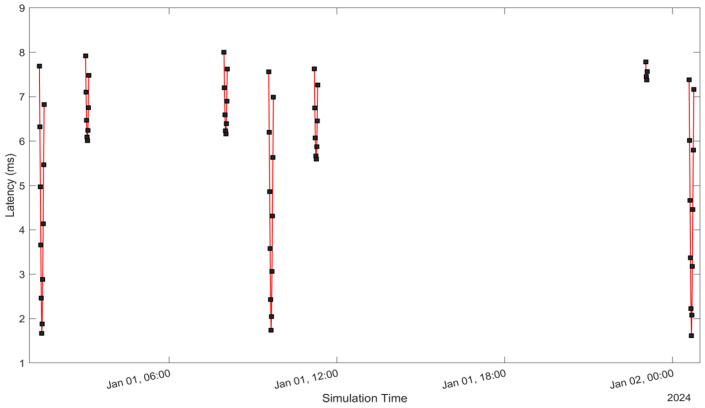
Latency of the ISS NAUKA satellite with initial satellite data.

**Figure 12:**
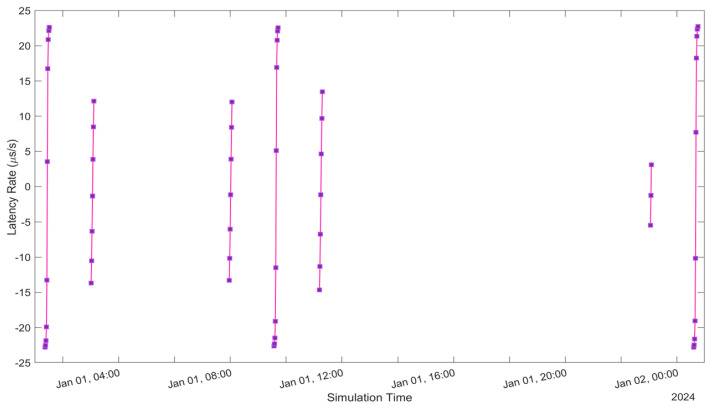
Latency rate of the ISS NAUKA satellite with initial satellite data.

See Figure 5, Figure 6, Figure 7, Figure 8, Figure 9, Figure 10, Figure 11 and Figure 12. Figure 5 and Figure 9 illustrate the Doppler shift, Figure 6 and Figure 10 show the Doppler rate, Figure 7 and Figure 11 describe latency, and Figure 8 and Figure 12 discuss the latency rate corresponding to the Globalstar and ISS with initial satellite data. The original Doppler shift possesses a dynamic range deemed too vast for effective training with Mean Squared Error (MSE) loss. By converting the values to a normalised scale, our proposed model achieved improved training precision and accuracy. We have optimised the training process to minimise the error for enhanced performance.

(15) $$\mathrm{Loss}=\frac{1}{N}\sum_{g=1}^{N}{\left|DS_{g}-{\tilde{DS}}_{g}\right|}^{2}$$

In the context of our work, $DS_{g}$ represents the normalised ground truth Doppler shift, while $-{\tilde{DS}}_{g}$ denotes the estimated normalised Doppler shift for sample g within the total N training samples.

**Figure 13:**
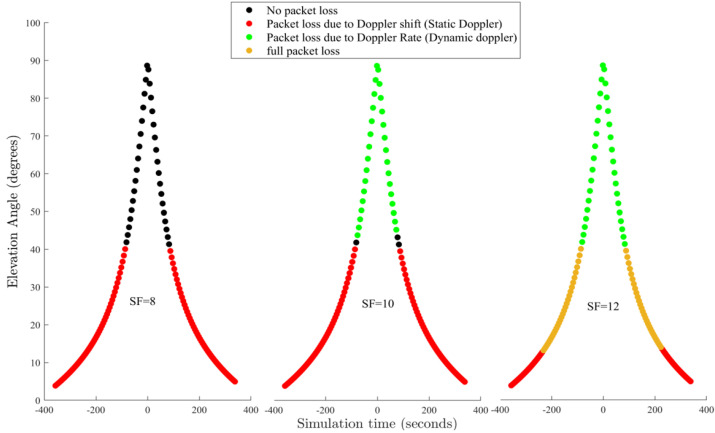
Effects of varying spreading factors (SFs) on LoRa performance of the Globalstar satellite. Simulation started on 1 January 2024 for 24 h, with simulation steps executed every second. Bandwidth is set at 500 MHz, Carrier frequency range is between 5091 to 5250 MHz, Application payload is set at 55.

In contrast, the LSTM algorithm serves as the learning model, demonstrating proficiency in comprehending and forecasting patterns. Figure 13 shows the effects of varying spreading factors (SFs) on LoRa performance. Simulation started on 1 January 2024 for 24 h, with simulation steps executed every second. The bandwidth is set at 500 MHz, the carrier frequency range is between 5091 to 5250 MHz, and the application payload is set to 55.

### 2.5. Impact of Doppler on LoRa Satellite

The study continues to comprehensively analyse the impact of the Doppler effect on the dependability of LoRa satellite connections and determine the latency and latency alteration calculation. We evaluated the occurrences of packet losses by differentiating between Doppler rate and shift. The Doppler rate is due to fluctuations in the velocity of satellites in LEO about an Internet of Things (IoT) device situated on the ground [33].

The study also determines the propagation delay from the LEO satellites to the Australian ground stations and assesses the propagation delay rate of change. After that, we graph the propagation delay linked to the initial satellite. While it is possible to graph the propagation delay for all satellites, Figure 10 and Figure 12 showcase the initial satellite to provide clarity and streamline the visualisation. Furthermore, it involves evaluating the rate of change of the propagation delay for both satellites also shown in Figure 10 and Figure 12. Subsequently, we plot the propagation to enhance clarity and simplify visualisation.

Our comprehensive analysis encompasses a range of communication variables and configurations, such as bandwidth and carrier frequency and has successfully pinpointed the necessary parameters for establishing direct connections to low-Earth-orbit (LEO) satellites utilising LoRa technology. These results can be leveraged to guide the selection of ideal parameters for upcoming system designs.

Specifically, our study demonstrates that the packet delivery rate for the most sensitive spreading factor (SF12) exceeds 91% with 11.5 MHz bandwidth for Globalstar and 145.8 MHz for ISS NAUKA, a carrier frequency of 631 MHz for the ISS located at an altitude of 402.3 km and 500 MHz for Globalstar at an altitude of 1414 km.

### 2.6. Satellite Link Budget Perspective

**Figure 14:**
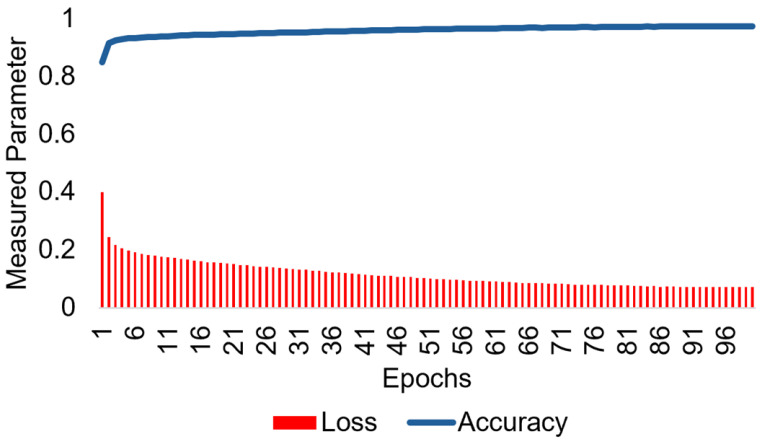
Mean square error loss is shown in red and accuracy in blue for the estimated Doppler shift MHz of the proposed ISS-LSTM.

We used the Satellite Link Budget Analyser application of MATLAB to comprehensively analyse link budgets for SatCom systems as shown in Table 3. A few key features included were:

- Conducting a comprehensive analysis of link budgets by inputting precise parameters related to the geographical location, characteristics of LEO satellites and ground stations in Australia, as well as atmospheric conditions such as rain attenuation(dB), total atmospheric losses (dB), and total propagation losses (dB) that may affect the connection. Figure 14 shows the loss and accuracy in red and blue for the ISS LSTM model.
- Creating a Globalstar and ISS communication link that satisfies a predetermined minimum link margin threshold.
- Utilising detailed calculations for interim link budget assessments.
- Performing evaluations, computations, and visual representations of results while considering various design constraints.

**Figure 15:**
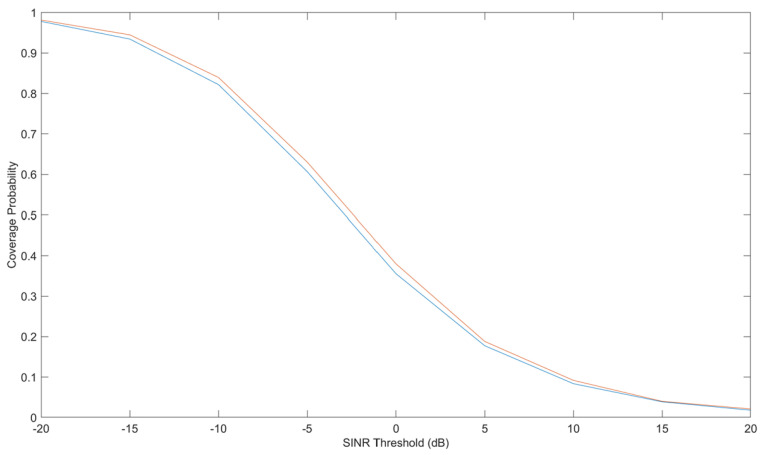
Coverage probability of uplink with signal-to-noise-and-interference-ratio (SINR) threshold in decibels on the *X*-axis. The different color lines show the Coverage probability of uplink with the SINR threshold in decibels.

**Figure 16:**
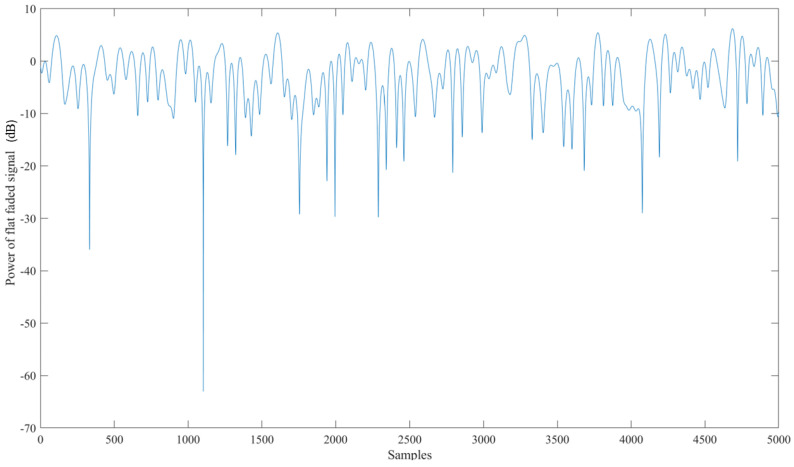
Power of the flat faded signal corresponding to the number of samples taken.

**Figure 17:**
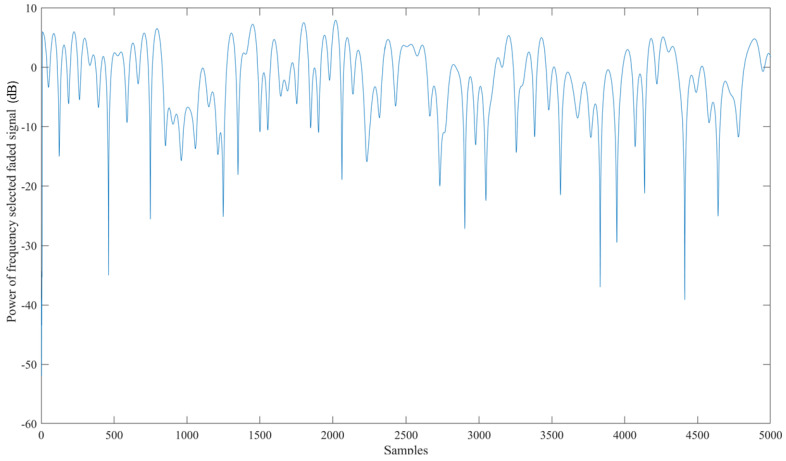
Power of the frequency and selected faded signal corresponding to the number of samples taken.

An exhaustive examination and simulation of ground stations to ascertain the Signal-to-Noise-and-Interference-ratio (SINR) and Coverage probability was performed for both downlink and uplink scenarios. The Globalstar satellite features a complex communications infrastructure, including S- and L-band antennas, a trapezoidal body, and two solar arrays. Each satellite operates at an altitude of 1414 km, equivalent to approximately 876 miles above sea level. Figure 15 shows the Coverage probability of uplink with the SINR threshold in decibels on the *X*-axis. Figure 16 shows the power of the flat faded signal and frequency selected faded signal on the *Y*-axis in Figure 17.

The following chart displays the outcomes of the calculated link budget analysis for assessing the availability of the SatCom link using the Earth–Space Propagation atmospheric loss model as outlined in Recommendation ITU-R P.618-13 [34].

## 3. Results and Discussion

Our study involved conducting thorough simulations to analyse the computational complexity, scalability, and mean squared error performance of deep learning architectures in comparison to traditional signal processing techniques. The findings show that training with quantised data obtained from signals with signal-to-noise ratios (SNRs) between 0 and 30 dB significantly boosts the performance of deep learning estimators across the entire range of SNRs examined.

We effectively detected and tracked the trajectories of two LEO satellite constellations using ground-based radar systems and conducted calculations for latency and Doppler in a satellite setting utilising LoRa modulation and analyzing suitable resolutions. Our initial assignment entailed the creation and upkeep of a comprehensive inventory of celestial bodies circling Earth, a vital component in space surveillance. The process involved the detection and categorisation of newly discovered objects, the inclusion in the inventory, the maintenance of accurate orbital information for existing objects, the continuous monitoring of orbit alterations over time, and the forecasting of potential reentries into the Earth’s atmosphere. These tasks are essential to ensuring the safety of operations in outer space and mitigating the risk of collisions with other satellites or established debris.

In investigating the LoRa ground SatCom link budget and scalability, while considering several channel conditions and user densities that mirror realistic application scenarios, it has been determined that LoRa modulation can establish a connection with an LEO. This can happen with a huge percentage of success of more than 97% at a distance of roughly 2300 km. Additionally, it has been found that the network can accommodate extensive connectivity. The mid-850 MHz band is less preferable than the 500 MHz band due to increased susceptibility to the Doppler effect. The significance of the lower band lies in its widespread coverage in various global regions and its proximity to less than 930 MHz band, which are crucial for coverage in countries of the northern hemisphere. This underscores the importance of the study conducted as ISS NAUKA has a downlink frequency of 631 MHz and Globalstar 500 MHz. The analysis also suggests that higher Spreading Factors, such as SF12, are more susceptible to a Doppler shift when compared to lower spreading factors in a mobile environment. As the bandwidth B decreases in Figure 10, the LoRa modulation becomes increasingly susceptible to static Doppler effects. This susceptibility is illustrated in Figure 10. The legend’s colour coding effectively differentiates between correct reception with the black dots and red, green, and yellow dots indicating Doppler rate, shift, or a combination. Upon observation, it is noted that there is a loss of data packets when the elevation angle (E) is equal to or less than 40 degrees, regardless of the spreading factor (SF) utilised. Unlike SF8, which functions effectively for E angles greater than 40 degrees, dynamic Doppler affects both SF 12 and 10, which are characterised by fluctuations in frequency during the reception of data packets due to the changing Doppler shift rate. Our findings suggest that static Doppler interference poses challenges at lower altitudes, whereas dynamic Doppler interference proves more effective as the satellite approaches the target at higher altitudes. This phenomenon is linked to a reduction in the satellite’s coverage range, leading to a gap in communication. As a result, the window of reliable Doppler connection decreases, potentially impeding the establishment of device communication. Our analysis includes assessments for modulation techniques commonly used in Internet of Things (IoT) standards, such as long-range (LoRa) modulation. It is worth mentioning that higher elevation angles result in a higher Doppler rate (absolute value). Specifically, SF10 is susceptible to the dynamic Doppler effect for E angles greater than 40 degrees, while SF12 begins to experience difficulties at E angles exceeding 13 degrees.

In comparison to ground objects, LEO satellites induce a considerably greater Doppler shift as they navigate around the Earth at an average velocity of thousands of kilometres per hour. Given the substantial dynamics inherent in an LEO satellite, Doppler positioning algorithms are poised to reap significant advantages from this heightened dynamic attribute. Through our innovative approach, we have successfully demonstrated the efficacy of accurately estimating carrier frequencies from 1-bit quantised data with reduced reliance on pilot signals and lower SNRs when compared to conventional signal processing methodologies. We rigorously analyze and simulate ground stations to ascertain the signal-to-noise-and-interference ratio (SINR) and coverage probability for downlink and uplink scenarios.

**Figure 18:**
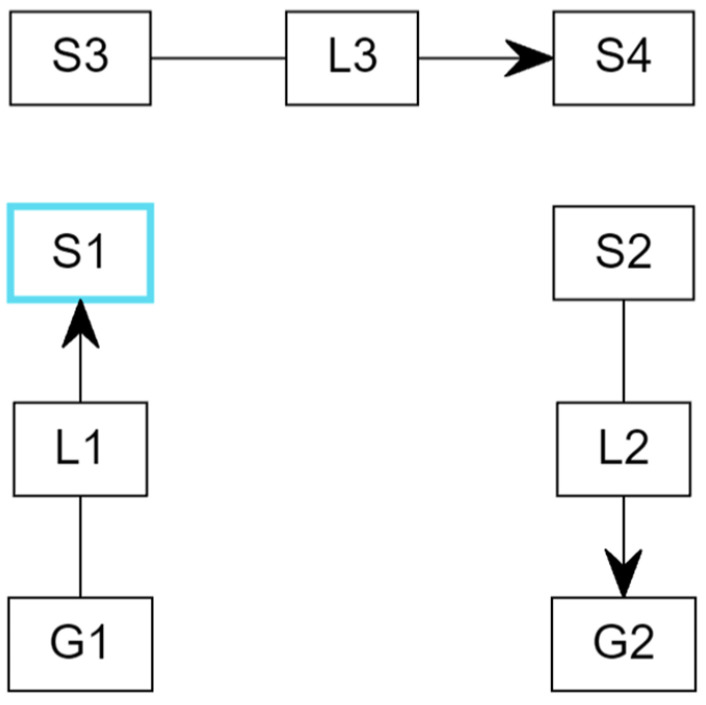
Configuration for the Satellite Link Budget for the Globalstar satellite and the Perth ground station. Link L1 acts as an uplink enabling a connection between ground station G1 and satellite S1. Link L3 operates as a critical intermediary, facilitating communication between satellites S3 and S4. Link L2 functions as a downlink, enabling communication between satellite S2 and ground station G2.

The Globalstar satellite is equipped with a sophisticated communications infrastructure including S- and L-band antennas, a trapezoidal body, and two solar arrays. Each satellite operates at an altitude of 1414 km, approximately 876 miles above sea level. The ISS maintains an orbital altitude range of 370–460 km (200–250 nautical miles) above the Earth’s surface. The ISS orbital inclination is set at 51.6 degrees, enabling the station to traverse over 90% of the populated regions on Earth. Figure 18 illustrates the configuration for the Satellite Link Budget configuration for the Globalstar satellite and the Perth ground station as follows:

- Link L1 acts as an uplink, enabling the establishment of a connection between ground station G1 and satellite S1.
- Link L3 operates as a critical intermediary, facilitating communication between satellite S3 and satellite S4.
- Link L2 functions as a downlink, enabling the flow of communication between satellite S2 and ground station G2.

Summarising the above discussion, the classification challenges outlined in this study are critical for optimising throughput and reducing bit error rates. The machine learning and deep learning algorithms examined in this context are specifically designed to address classification tasks and aim to improve the operational efficiency of low Earth orbit (LEO) satellites in the context of the Doppler effect. A thorough analysis has been performed regarding the performance of LoRa and ground satellite connectivity utilising machine learning and deep learning techniques, with particular attention given to the substantial impact of the Doppler weather effect.

The hybrid artificial intelligence model that has been developed demonstrates a high degree of effectiveness in classifying extended transmission distances, albeit with a consideration of bandwidth limitations. Additionally, the AI framework is capable of predicting the Doppler profile for low-Earth-orbit (LEO) satellites, while also incorporating factors related to weather attenuation. This integrated approach serves to optimise the project for both uplink and downlink transmission processes. As a result, there is a significant enhancement in computational efficiency and overall system performance. Furthermore, this methodology allows for comparative analyses against other prominent modulation schemes that are relevant within the Internet of Things (IoT) standards, including the Doppler effect associated with long-range modulation.

The classification analysis has focused on several key factors that differentiate the impacts of static Doppler from those of dynamic Doppler. Furthermore, the challenges presented by the Doppler effect in establishing LoRa ground satellite connectivity have been comprehensively investigated. This classification framework provides valuable insights into identifying the most suitable parameters for satellite communication, thereby enhancing our understanding of packet loss dynamics.

**Figure 19:**
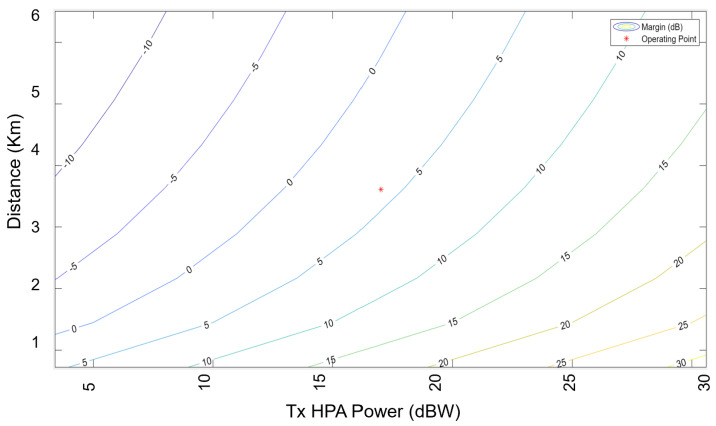
Margin and operating point of Globalstar satellite and Perth ground station.

**Figure 20:**
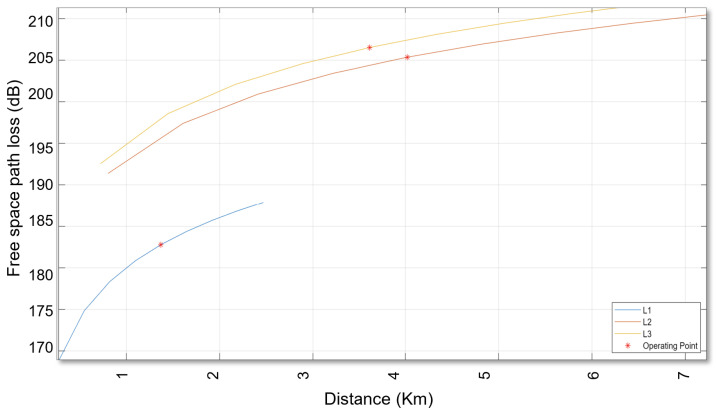
Free space path loss in Decibels for the Globalstar satellite and the Perth ground station.

Furthermore, it is important to note that the computational demands and simulation duration are largely influenced by the parameters being studied. For example, using MATLAB R2023a requires around 20 s to generate the outcomes shown in Figure 19 and Figure 20. Additionally, it should be emphasised that resistance to the Doppler impact greatly depends on the decisions made by the designer, and the overall efficiency has a better chance to increase with design advancements.

## 4. Conclusions

Efficient protocols are essential for ensuring reliability, smooth communication, and energy efficiency. Through cutting-edge AI architectures and emerging technologies, robust satellite communication networks are feasible.

This study comprehensively examines the operational efficiency of low-Earth-orbit satellites concerning the Doppler effect, utilising state-of-the-art artificial intelligence techniques. The performance of LoRa and ground satellite connectivity has been thoroughly investigated, particularly under the influence of a significant Doppler weather effect. The hybrid AI model developed prioritises extended transmission distances, even at the cost of bandwidth.

This study estimates frequency offset, uses AI to project the Doppler profile for the LEO satellite and weather attenuation, coordinates the project collectively for the uplink and downlink, enhances computational efficiency and overall performance, and assesses against other prominent modulation schemes found in prevalent Internet of Things (IoT) standards such as Doppler effect in long-range modulation. In LEO satellite communications, the implementation of effective frequency Doppler shift (FDS) compensation techniques is vital to ensure optimal data transmission rates and minimal error occurrences. The analysis has focused on several key factors that differentiate the impact of the static Doppler from the dynamic Doppler. The challenges posed by the Doppler effect when establishing LoRa ground satellite connectivity have also been addressed.

The meticulous evaluation of the traditional systems with the current industry benchmarks helps us to refine modulation schemes, especially in the long range (LoRa), leading to a significantly improved and more effectively operated AI model. With training and optimisation of the model developed in conjunction with SNR values, we can enhance estimation accuracy and reduce computational complexity. It has been observed that various factors, such as spread factor, frequency offset, and bandwidth have a significant effect on the uplink. Furthermore, the satellite’s height of orbit and packet payload also have a moderate effect on performance. These conclusions can help in determining the most suitable parameters for SatCom to understand the loss of packets.

Our study highlights the benefits, challenges, and delicate equilibrium in adjusting key parameters related to communication. The presence of both Doppler rate and shift effects could interfere with the SatCom link, potentially resulting in the loss of packets. Static Doppler is challenging at elevation angles that are lower. However, dynamic Doppler has a more significant effect at elevation angles that are higher, especially near the end device. These effects can result in coverage gaps within the impression of the satellite, decreasing the area of coverage, where it is impactful and reduces the available time for a stable connection. As a result, even if a satellite is within sight, other systems may encounter difficulties establishing communication due to the influence of the Doppler effect. The integrated AI model, known as the Iterative Input Selection–Long Short-Term Memory Network (IIS-LSTM), forecasts Doppler weather profiles for LEO satellites. The IIS algorithm is utilised for selecting pertinent input variables within the model, whereas the LSTM algorithm is responsible for acquiring knowledge and making predictive assessments of patterns. The feature extractor is trained using the normalised mean squared error criterion to accommodate the wide range of Doppler shift values present in LEO communication channels.

The feature extractor has been trained utilising the normalised mean squared error criterion in order to adapt to the diverse range of values of Doppler shift encountered within LEO. The machine learning model utilising XG Boost attains an impressive accuracy rate of 87%, with the 55th trial emerging as the most optimal out of a total of 100 trials. Additionally, the learning rate employed in this analysis is 0.102. It is observable that the XGB model demonstrates efficiency in the testing duration, achieving approximately 0.0997 s for elevated signal-to-noise ratios and delivering an accuracy of 87%. Nonetheless, it is noteworthy that this model incurs significant computational costs.

In comparison, the IIS-LSTM model showcases a commendable accuracy of 87% while offering improved computational efficiency for lower SNRs at around 0.4651 s. Following closely are the XGB and CNN models, with respective testing times of approximately 0.5990 and 0.6120 s. Additionally, as the number of channel epochs increases, the estimation performance improves. However, it is important to consider that increasing the number of epochs may not always be beneficial, particularly in low-SNR conditions where the estimation error may converge.

Specifically, our study demonstrates that the delivery rate of the packet for the spreading factor (SF12) is more than 91% with 11.5 MHz bandwidth for Globalstar and 145.8 MHz for ISS NAUKA, and the carrier frequency of 631 MHz for ISS located at an altitude of 402.3 km and 500 MHz for Globalstar at an altitude of 1414 km. The technique developed has the potential to address high orbital signal fluctuations. Our innovative model enhances the channel for capturing features that are high-dimensional and accurately measuring the Doppler shift. This approach shows promising results and has the potential to significantly enhance the estimation of the Doppler shift in LEO satellites. By leveraging sophisticated hardware architectures and cutting-edge platforms, the establishment of expansive networks can be facilitated through the deployment of efficient transmission technologies such as LoRa. Central to this process are protocols and network management strategies that prioritise reliability, transmission range, and energy efficiency. These methodologies all aim to improve robustness and increase transmission distances, albeit at the expense of certain satellite operational factors. Furthermore, it is imperative to carefully consider the integration of network management solutions and seamless incorporation into existing IoT frameworks to address the complexities and opportunities in designing, developing, implementing, and deploying wireless low-power and long-range sensors and networks comprehensively.

## 5. Limitations and Future Research Work

Despite progress in the field, there are several obstacles and outstanding concerns regarding the effective deployment of these technologies on a widespread basis. In circumstances involving vast areas, remote or inaccessible locations, or hazardous environments, the capability to enable reliable communication without depending on current infrastructure offers a substantial benefit of low-power and long-range technologies.

It is imperative to develop a comprehensive strategy for future LEO connectivity that provides a detailed outline of both the system and technology components involved in space networking and communications. Additional enhancements may be incorporated into the existing study analysis outlined within this document. Implementing AI techniques for satellite communication parameters optimisation is key to developing efficient low-power, long-range network infrastructures for the future. It is also important to consider comprehensive network management strategies and the seamless integration of these networks into existing IoT ecosystems in this rapidly changing environment. By taking advantage of the robust signal reception of LEO satellite signals and leveraging the radio environment-reshaping capability of RISs, the integration of these two cutting-edge technologies paints a compelling picture of a future where localisation services exceed the current limitations. Given the intricate computational demands associated with beamforming designs for reconfigurable intelligent surface (RIS) units, it is prudent to explore future studies about energy-efficient methodologies that incorporate artificial intelligence (AI) augmentation. Analyzing LoRa Doppler with noise and sensitivity presents a promising opportunity for future exploration. It is essential to recognise the lack of a specific approach for addressing the Doppler shift and rate, requiring additional research. A noteworthy consideration in the deployment of micro Doppler radar systems is the implementation of a federated learning strategy. Current research efforts, including those conducted by the authors [35,36], are actively exploring this area. It is proposed that a unified convolutional neural network (CNN) model could be employed across all distributed radar units (referred to as clients). This model would be trained utilizing a federated learning approach, leveraging a wireless backhaul connection to the central server in future investigations.

Another future direction can be developing solutions for a challenging situation when satellites are moving in opposite trajectories, resulting in the highest achievable Doppler shift. This scenario has the potential to create difficulties in sustaining consistent satellite communication, potentially resulting in data loss or system malfunction. Therefore, future research needs to focus on developing strategies to mitigate this Doppler shift and guarantee reliable communication.

## Figures and Tables

**Table 1:** The geographic description of the study sites.

City, State	Longitude (°E)	Latitude (°S)	Elevation (m)
Perth, Western Australia	115.86	31.95	32
Brisbane, Queensland	153.02	27.47	31.5

**Table 2:** Optimal architecture (in RED for *IIS-LSTM*).

Model	Hyper-Parameters	Optimal Set Grid Search
*IIS-LSTM*	Epochs Activation function Batch size Optimiser, Drop rate *IIS-LSTM* filter Dropout SNR Platform	[5, 10, 50, 100, 200] [SoftMax, *Tanh*, *ReLU*, Leaky *ReLU*, *sig*] [100, 500, 700, 1000, 2000] [*Adam*], [0.1, 0.2] [50, 60, 100, 200, 500] Yes [0–30] Python (version 3.9)
*CNN*	Epochs Activation function Batch Size Optmiser, Drop rate *CNN* filter Pooling, Padding Number of Dense layers used Dropout SNR Platform	[5, 10, 50, 100, 200] [SoftMax, *tanh*, *ReLU*, Leaky_*ReLU*, *sig*] [100, 500, 700, 1000, 2000] [Adam ], [0.1, 0.2] [50, 60, 100, 200, 500] [same] [3 for Input and 1 for Output] Yes [0–30] Python (version 3.9)
*XGBoost*	Gamma Maximum Delta Step Lambda Alpha Weight Seed max depth Platform	[Tuned with loss function] [No] [0.5–1] [used for reducing overfitting] [used for high dimensionality] [>0] [0] [3–10] *MATLAB* R2023b

**Table 3:** Link budget analysis for assessing the availability of the Geostar LEO satellite using the Earth–Space Propagation atmospheric loss model.

Tag	Name	L1	L2	L3
N1	Distance (km)	$1.2426\times 10^{4}$	$4.0215\times 10^{4}$	$3.6138\times 10^{4}$
N2	Elevation (deg)	−75.6539	13.5231	41.5653
N3	Tx EIRP (dBW)	32	46	47
N4	Polarisation loss (dB)	3.0103	3.0103	3.0103
N5	FSPL (dB)	181.9385	205.3634	206.5296
N6	Rain attenuation (dB)	-	13.0271	0
N7	Total atmospheric losses (dB)	-	15.2073	0
N8	Total propagation losses (dB)	181.9385	220.5707	206.5296
N9	Received isotropic power (dBW)	−155.9488	−180.5810	−165.5399
N10	$C/N_{0}$ (dB-Hz)	95.6503	71.0181	86.0592
N11	C/N (dB)	25.0433	3.2366	18.2777
N12	Received $E_{b}/N_{0}$ (dB)	25.6503	1.0181	16.0592
N13	Margin (dB)	13.6503	−10.9819	4.0592

## Data Availability

The original contributions presented in the study are included in the article, further inquiries can be directed to the corresponding author.

## Abbreviations

The following abbreviations are used in this manuscript:

AI	Artificial Intelligence
SatCom	Satellite Communication
LEO	Low-Earth orbit
IoT	Internet of Things
ISS	International Space Station
IIS-LSTM	Iterative Input Selection—Long Short-Term Memory Network
CNN	Convolutional Neural Network
XGBoost	Extreme Gradient Boosting
LoRa	Long Range
CSS	Chirp spread spectrum
ACF	Autocorrelation coefficient function
LFM	Linear frequency modulation
AWGN	Additive white Gaussian noise
MIMO	Multiple-Input Multiple-Output
DNN	Deep neural network
GRU	Gated recurrent unit
RNN	Recurrent neural network
HAPS	high altitude platform station
DL	Deep learning
LPWAN	Low-Power Wide-Area Network
SGP4	Simplified General Perturbations-4
JPDA	Joint Probabilistic Data Association
TLE	Two Line Element File
MEO	medium Earth orbit
GEO	geostationary
SNR	signal-to-noise ratio
OFDM	Orthogonal Frequency Division Multiplexing
BPSK	Binary Phase Shift Keying
CPI	coherent processing interval
GPU	Graphics Processing Unit
CPU	Central Processing Unit
